# Selective Attention and Inhibitory Control of Attention Are Correlated With Music Audiation

**DOI:** 10.3389/fpsyg.2020.01109

**Published:** 2020-06-03

**Authors:** Noemí Grinspun, Luc Nijs, Leonie Kausel, Kelsey Onderdijk, Nicolás Sepúlveda, Antonio Rivera-Hutinel

**Affiliations:** ^1^Departamento de Música, Universidad Metropolitana de Ciencias de la Educación, Santiago, Chile; ^2^Institute for Psychoacoustics and Electronic Music, Ghent University, Ghent, Belgium; ^3^Centro de Investigación en Complejidad Social, Facultad de Gobierno, Universidad del Desarrollo, Santiago, Chile; ^4^Departamento de Psicología, Facultad de Ciencias Sociales, Universidad de Chile, Santiago, Chile; ^5^Instituto de Entomologia, Universidad Metropolitana de Ciencias de la Educación, Santiago, Chile

**Keywords:** musical abilities, audiation, executive function, selective attention, inhibitory control of attention

## Abstract

Executive functions (EFs) are cognitive functions needed for adaptive and targeted behavior. Music aptitude is the potential or capacity for musical achievement. A key element of music aptitude is audiation, defined as the process through which sound becomes music and meaning is attributed to that music. In this paper, we report on the association between audiation skills and executive skills. Not only is this important to consider the validity of the audiation tests, but also to better understand the concept of audiation and its link to cognitive skills. We conducted an empirical study, in which a sample of second grade school students from two elementary schools, one from Ghent, Belgium (*N* = 36) and the other from Santiago, Chile (*N* = 25), were administered both a musical aptitude and an attention and inhibitory control test. We hypothesized that a positive correlation exists between sustained attention, inhibitory control and music aptitude.

## Introduction

More than ever, music is ubiquitous in our daily lives. Even before birth we are often presented with music in our environment and this has an impact on children’s development. Indeed, research indicates that early auditory experiences influence human development at different levels, especially since the fetus experiences sounds in an emotional, multimodal and vibro-acoustic manner during development (Haslbeck and Bassler, 2018). For example, neuroscientific research shows that music not only affects the development of our brain (e.g., limbic and paralimbic structures) but also enhances psychological and physiological health (Koelsch, 2010, 2014). As such, some scholars argue that musical learning starts even before birth (Huotilainen and Näätänen, 2010; Perani et al., 2010).

In addition to environmental stimuli, the developing fetus is presented with endogenous stimuli emerging from the mother’s body and behavior. According to Teie (2016), who argues in favor of a match between the universal musical features and the sounds in the womb, the fetal acoustic environment provides the base for the fundamental elements that are found in music. Arguably, due to exposure to music in prenatal life and due to the endogenous presentation of basic musical features in the womb sounds, babies are not only born with for example language skills but also with a range of musical abilities or potential musical achievement, also called “music aptitude”(Gordon, 2007).

Although, according to Gordon, a child is born with this potential, it’s realization is highly dependent on the environment and the early musical experiences of the child. Until the age of nine, music aptitude is rather dynamic and fluctuating due to environmental factors (Gordon, 2007). Therefore, both exposure to music in early life and music instruction can play an important role in realizing the musical potential of a child. After the age of nine, one’s music aptitude becomes stabilized (Gordon, 2007).

In relation to music aptitude, Gordon coined a concept that has become seminal, namely the concept of audiation, referring to the process through which sound becomes music and meaning is attributed to that music. Originally defined as “hinged mosaic relationships linked to networks of comparative pattern structures” (Gordon, 2017, p. 6), audiation or “thinking music in the mind with understanding” is based on the assimilation and comprehension of the sounds one is hearing (Dalby, B:2)^1^. As such, it involves concentrating on one set of musical sounds (Sportsman, 2011; Gordon, 2007). Audiation is fundamental to music aptitude and, consequently, to music achievement (Gordon, 2007).

Gordon (2007) proposes that, when we listen to familiar or unfamiliar music, we go through six music-processing steps as we audiate. First, the auditory information is briefly kept in sensory memory (in response to sound). Second, tonal and rhythmic patterns are audiated and a tonal center and macrobeats are identified. Third, the musical context is audiated based on the mental establishment of tonality and meter. Fourth, rhythmic and tonal patterns are structured and retained. Fifth, patterns that were previously audiated are recalled. Finally, tonal and rhythmic patterns are anticipated and predicted. These steps are continuously repeated. Gordon asseverates that the more one places music processing demands on the brain through formal/informal music instruction and musical activities, the more audiation skills will develop (Gordon, 2007).

Plausibly, all these processes are related to executive functions (EFs; Slevc et al., 2016), which was also proposed by Gordon (Sportsman, 2011). To our knowledge, this relationship has not been shown empirically.

Executive functions are a group of cognitive processes that are necessary for adaptive (social) behavior, such as goal-oriented tasks. They are related with a neural network in which one of the most important structure is the prefrontal cortex (Lezak et al., 2004; Blair et al., 2005; Diamond and Lee, 2011) and include the capacity of hold and change attentional focus, temporarily maintain the information [working memory (WM)], to organize the information, to self-monitor, inhibit responses, think flexibly, to plan the future actions (Huizinga and Smidts, 2011; Lesiuk, 2015), and also includes the ability to “deal with novelty” (Chan et al., 2008, p. 201). These processes are crucial for novel or complicated tasks, challenging for sustained attention (Miller and Cohen, 2001; Zelazo et al., 2003). As they influence almost every aspect of cognition (Diamond and Lee, 2011).

It has been difficult to define one way of evaluating EFs since most of the tests do not measure the same unitary construct (Packwood et al., 2011; Karr et al., 2018). According to many researchers, there are three interdependent EFs (Miyake et al., 2000; Lehto et al., 2003; Diamond, 2013): inhibitory control (selective attention, cognitive inhibition, and self-control), WM, and cognitive flexibility. Inhibitory control involves the ability to control attention, behavior, thoughts and/or emotions in order to cancel strong internal predispositions or external temptation, and instead act in a more appropriate way (Diamond, 2013). Without inhibitory control our impulses, old habits of thought or actions would take over (Diamond, 2013). There has been a variety of suggested inhibitory functions, one example is inhibition proposed to be applied in situations that involve a demand of resistance to interference from distracting or competing stimuli (Friedman and Miyake, 2004; Howard et al., 2014). Inhibitory control of attention allows us to selectively attend, focus on what we select and suppress attention to other stimuli (Posner and DiGirolamo, 1998; Theeuwes, 2010, see Diamond, 2013). As such, inhibition plays an important role in selective attention, i.e., the deployment of attentional focus on task relevant features of the environment. Furthermore it is important to sensory processing (Awh et al., 2000; Weiss et al., 2018), perception (Posner and Driver, 1992; Anderson and Ding, 2011; Weiss et al., 2018) and to performance on cognitively-demanding tasks (Posner and Rothbart, 2007; Gazzaley and Nobre, 2012; Weiss et al., 2018). Inhibitory control is related to WM, because in order to relate multiple ideas or get facts together one must be able to resist focusing only on one thing. To keep the mind focused, it is necessary to inhibit internal and external distractions (Diamond, 2013). Successful performance in creative and other settings have been predicted by differences in cognitive flexibility (De Dreu et al., 2008; Dennis and Vander Wal, 2010; Hirt et al., 2008; Lowrey and Kim, 2009; Adi-Japha et al., 2010; see Figueroa and Youmans, 2011). Inhibitory control is related with cognitive flexibility as well. One aspect of cognitive flexibility is being able to change perspectives. To change perspectives, it is necessary to inhibit previous perspectives and load others into WM or activate a different perspective, cognitive flexibility requires and builds on inhibitory control and WM (Diamond, 2013).

Cognitive flexibility has been defined as the capacity to adapt strategies of cognitive processing to new conditions. It is in that manner that cognitive flexibility is connected to attentional processes (Cañas et al., 2003; Moore and Malinowski, 2009).

Listening to music requires perceptual abilities such as pitch discrimination, auditory memory, to grasp the temporal and harmonic structure of the music as well as its affective components. This perceptual process engages a distributed network of brain structures (Peretz and Zatorre, 2005). Evidently, this requires refinement of selective attention skills and implicit learning of the acoustic and syntactic rules that structure musical sounds (Kraus and Chandrasekaran, 2010; Strait et al., 2010). Music processing not only needs memory and attention, but also the capacity to integrate discrete acoustic events into a coherent perceptual stream based on specific syntactic rules (Kraus and Chandrasekaran, 2010). Additionally, auditory WM and attention are found to be significantly related to rhythm perception ability (Strait et al., 2011). The auditory cognitive system depends on WM mechanisms in order to maintain a stimulus on-line to be capable of relating one element to another in a sequence. For music recognition, it is also necessary that the perceptual memory system accesses and selects potential predictions (Bella et al., 2003; Peretz and Zatorre, 2005).

It is also agreed that the ability to sustain attention voluntarily is supported by higher-order cognitive functions characterized as EFs, such as inhibition and WM.

Gordon theorized that in each of the six music processing steps, audiation is linked to EFs (Sportsman, 2011):

• *Momentary retention of auditory information:* directing attention to relevant auditory information and inhibiting attention to irrelevant stimuli, keeping auditory information in WM.
• *Audiation of tonal and rhythmic patterns and identification of tonal center and macrobeats:* holding tonal and rhythmic patterns in WM and selectively attending to particular aspects of the patterns.
• *Audiation of tonal context by grasping the tonality and meter:* updating WM with relevant tonal and metrical content while keeping patterns in mind.
• *Organization and retention of pattern:* retaining important patterns in WM and inhibiting attention to less relevant details.
• *Recall of previously audiated patterns:* retrieving and comparing previously audiated musical material, holding tonal and rhythmic patterns in WM, including their tonal and metrical context and the previously audiated material retrieved from WM.
• *Anticipation/prediction of tonal and rhythmic patterns*: thinking about the tonal and rhythmic patterns that, based on available information (current and past), are likely to occur next g. Attention is maintained through the music to see if predictions are satisfied. Repeatedly updating WM with new material and engaging in the six step cycle.

Following these considerations, we wanted to verify how EFs are related to music aptitude.

In this paper, we report on the association between audiation skills and executive skills. Not only is this important to consider the validity of the audiation tests, but also to better understand the concept of audiation and its link to cognitive skills.

We administered both an aptitude and an attention, inhibitory control and cognitive flexibility test. As this work is part of a study on children’s ability to synchronize with music, and considering that attention is significantly related to rhythm perception ability (Strait et al., 2011), we hypothesized that a positive correlation exists between music aptitude and selective attention and inhibitory control.

Furthermore, as this study involved a school in Chile and a school in Belgium, due to the collaboration between Chilean and Belgian researchers, we were interested to see whether this could differ according to the cultural context.

## Materials and Methods

### Participants

Sixty one second grade children (25 females, mean age: 7.4 years, SD = 0.52) participated in this study. All children were recruited from two elementary schools, one from Ghent, Belgium (demographics: 15 girls, mean age: 7.4, SD = 0.62, 21 boys, mean age: 7.4, SD = 0.69 parents educational level (years): 16.6, SD = 2.07), and the other from Santiago, Chile (demographics: 10 girls, mean age: 7.6, SD = 0.5, 15 boys, mean age: 7.5, SD = 0.49, parents educational level (years): 7.6, SD = 0.5). Twenty percent of the children from the Belgian school were of foreign origin, and did not speak Dutch with their parents. All the children from the Chilean school were Chilean and spoke Spanish with their parents. All participants answered an adapted short version of the Montreal Music History Questionnaire (Coffey et al., 2011) (Dutch version in Belgium, Spanish version in Chile), which inquired about their personal experience in music and dance performance. Answers showed that 16 children from Belgium played a musical instrument, and 4 children from Chile played a musical instrument.

### Ethical Approval

The study protocol was reviewed and approved by the Research Ethics Committee from the Faculty of Arts and Philosophy, Ghent University and from the University of Santiago, Chile N°: 314/2019. The study was carried out in accordance with the relevant guidelines and regulations and all procedures were in accordance with the statements of the Declaration of Helsinki. For all participants, written informed consent was given by parents/caregivers and oral assent was given by children.

### Experimental Design

#### Procedure

Children participated in two sessions. In each session, they solved the following standardized tests: the rhythm subtest of the primary measures of music audiation test (PMMA) and the neuropsychological d2 test. In Belgium, the sessions were carried out with all children in the classroom. In Chile, the sessions were carried out in groups of three for PMMA test and individually for d2 test in the teachers’ community room of the school (Supplementary Table S1).

#### Standardized Tests

##### Primary measures of music audiation

**FIGURE 1:**
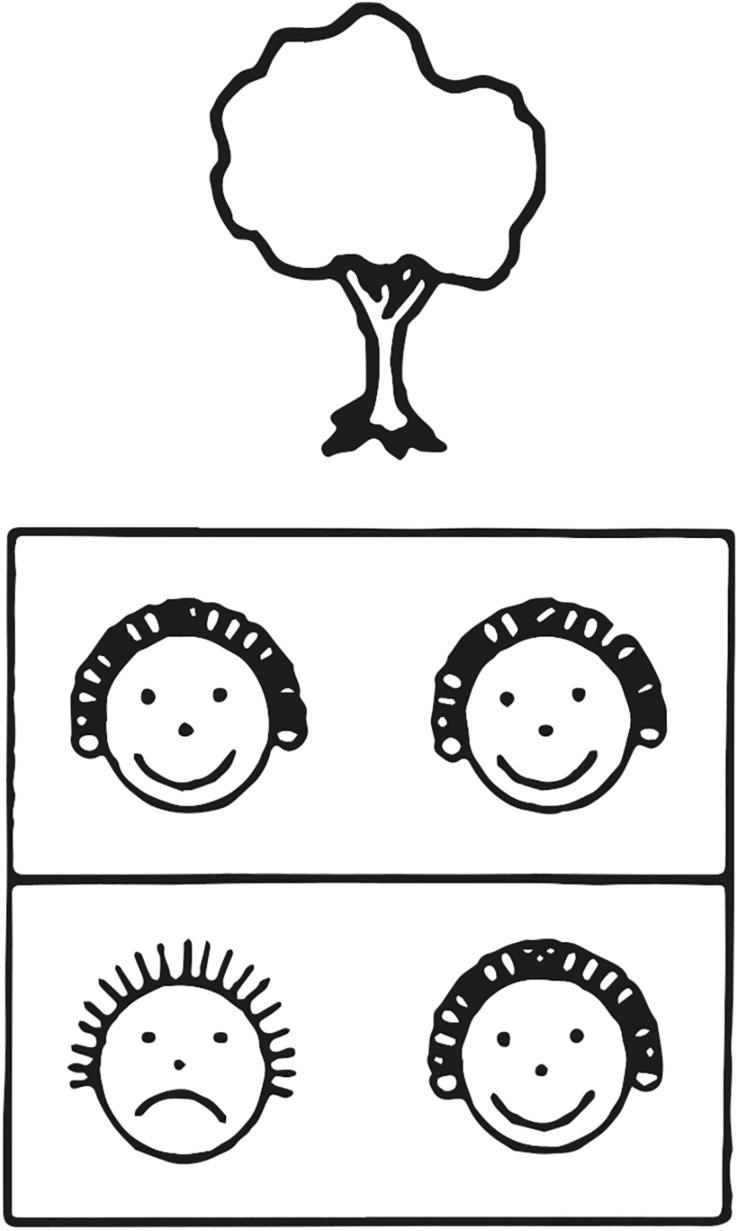
An example of a pictorial representation used in the PMMA. Each pair of rhythmic patterns has its own picture (e.g., tree or hand) and children draw a circle around the similar (laughing) faces (top) when they hear the two rhythmic patterns as sounding the same. They draw a circle around the dissimilar faces (bottom) when they hear the two rhythmic patterns as sounding different.

The rhythm subtest of the PMMA was used to measure music aptitude (Figure 1). The patterns of Gordon’s measures of audiation are carefully designed, whereby rhythmic patterns have no pitch variations. The rhythmic patterns are designed in both usual and unusual meters. A different timbre emphasizes the macro-beats. The test is designed for children from kindergarten (5–6 years old) to third grade (9–10 years old) and it was applied as described in Gordon, 1986. The reliability coefficient of the PMMA rhythm subtest (Gordon, 1979) is 0.86. This subtest presents 40 pairs of short rhythmic patterns, and children have to indicate whether the pairs contain two same or two different patterns by circling a pair of same or different faces on an answer sheet. The pause between the two presented patterns is four seconds, thereby not allowing for the first pattern to be compared through memorization but only recalled through audiation (Mazzotta, 2017). The total duration of the test is about 20 min. The rhythm sequences were presented with speakers at a comfortable listening level for participants.

##### d2 neuropsychological test

The d2 neuropsychological test was used to probe the children’s attention and inhibitory control (Brickenkamp, 1962, edition 2002), d2 test is a measure of selective attention and mental concentration, as well as impulsivity (Wassenberg et al., 2008).

This test has been proposed as a particularly useful measure of attention and concentration processes (Brickenkamp and Zillmer, 1998). The test is designed to be used in a wide age range (7–99 years). The psychometric properties of the d2 test have shown to be strong: (1) internal consistency, ranging between 0.95 and 0.98, and (2) a validity coefficient of 0.47 (Bates and Lemay, 2004; Brickenkamp et al., 2013). Moreover, the d2 test correlates highly with other measures of attention and is well validated (Brickenkamp, 2002) reliability (*r* > 0.90) (Steinborn et al., 2017). Furthermore previous research has shown that the Stroop-test correlates significantly with the TOT (*r* = 0.34) and CP (*r* = 0.34) scores of the d2-test (Brickenkamp and Zillmer, 1998; Moore and Malinowski, 2009).

**FIGURE 2:**
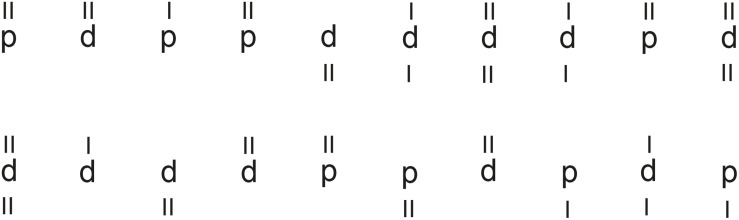
An example of d2 test. Every letter d or p is accompanied by little dashes above, below or both. The children have to mark with a/all the letters d accompanied by two dashes.

The test consists of 14 test lines with 47 characters in each line. Each character is the letter “d” or “p” marked with one, two, three, or four small dashes. The template contains a total of 299 relevant elements, with 45% of all elements having two dashes. The task is to scan the lines and cross out all occurrences of the letter “d” with two dashes while ignoring all other characters (Figure 2). This must be done in a time limit of 20 s per row. Pauses between rows are not allowed. The total duration of the test is between 8 and 10 min.

#### Data Analysis

The behavioral measures from the PMMA rhythm subtest include correct responses, which are classified in different percentiles according to accuracy. These are classified by age in a BAREMO table.

The behavioral measures from the d2 test include:

• TOT: Difference between the total number of items processed and the total number of errors, giving a quantitative measure of processing speed, attention, and inhibitory control (Moore and Malinowski, 2009; Lozano et al., 2015).
• CP: Difference between the number of correct responses and the number of commission errors (E2). It is considered as a concentration index, providing an index of coordination of speed and accuracy of performance.
• VAR: fluctuation rate, indicating sustained attention.
• E: Sum of all mistakes.
• E1: Errors sum of omission: a common mistake, sensitive to attentional control, rule compliance, and performance quality.
• E2: Errors of commission: a less common error, associated with inhibitory control, rule compliance, and cognitive flexibility.

All these measures are classified in different percentiles and classified by age.

#### Statistical Analysis

All behavioral data were studied using SigmaPlot 12.0 (SPSS Inc. Chicago, IL), except for the Principal Component Analysis (PCA) and the covariance analysis (ANCOVA), which was done under IBM SPSS^®^ 22.0 (IBM Corp., Armonk, NY). First, to analyze the main effect and interactions between geographic location, gender and musical background in accuracy on the d2 and PMMA tests we did a three-way analysis of variance (ANOVA), we applied Bonferroni correction for *post hoc* multiple comparisons. Then we evaluated the collinearity between CP, TOT, VAR, and Errors, defining the maximum VIF as 4 and the minimum tolerance as less than 0.2 (Hair et al., 2010). Subsequently, as we found correlations between the d2 behavioral measures, and to reduce their dimensionality, while preserving as much statistical information as possible, we conducted a PCA analysis. The three new components are linear functions of the originals (CP, TOT, VAR, and Errors) and are uncorrelated with each other. Then the new components were included in a ANCOVA with geographical location, musical background as fixed factors, and PMMA as a dependent variable. Statistically significant differences were considered at a *p*-value < 0.05. All data are expressed as mean ± s.e.m.

## Results

### Group Differences

To check for differences associated with the context, first we performed a three way ANOVA to investigate whether gender, musical background and geographical location are associated with the results of the d2 test, and PMMA. For gender, no significant differences were found for CP (*F* = 2.951; *p* = 0.100), TOT (*F* = 1.100; *p* = 0.299), Errors (*F* = 2.304; *p* = 0.135), VAR (*F* = 1.296; *p* = 0.260), and PMMA (*F* = 1.301; *p* = 0.259). For musical background, we did not find differences for CP (*F* = 1.352; *p* = 0.250), TOT (*F* = 0.558; *p* = 0.776), Errors (*F* = 0.573; *p* = 0.452), VAR (*F* = 2.411; *p* = 0.158), and PMMA (*F* = 0.603; *p* = 0.441). Only for geographical location we found significant differences for CP (*F* = 13.282; *p* < 0.001), TOT (*F* = 9.818; *p* = 0.003), and PMMA (*F* = 6.594; *p* = 0.002). No differences were found for Errors (*F* = 0.569; *p* = 0.455) and VAR (*F* = 1.841; *p* = 0.301). In addition, no interaction effects were found between gender, music background, and geographic location. These analyses confirm that neither gender nor musical experience had an influence on the results of the d2 and the PMMA. However, geographical location seemed to influence the d2 behavioral measures TOT and CP, and on PMMA.

### Musical Aptitude as a Function of Attention and Inhibitory Control

Initially, we conducted a multiple linear regression to model musical aptitude (PMMA percentile) as a function of CP, TOT, VAR, and Errors. However, as we found a collinearity between the variables CP and TOT (CP, Tolerance = 0.11, VIF = 8.53; TOT, Tolerance = 0.16, VIF = 6.20) we decided to exclude the variable TOT. Then, due to the results of the correlation analysis (see Table 1), and to further investigate this in-depth and to see whether there could be an effect of the co-variables, we performed a PCA analysis to reduce their dimensionality. Bartlet’s test of sphericity was highly significant at *p* < 0.001, indicating that the data were appropriate for PCA. The new components are linear functions of the originals (CP, TOT, VAR, and Errors) and now they are uncorrelated with each other. Principal component analysis generated a solution where one factor PC1 accounted for 62.6%, PC2 for 25.3% and PC3 for 11.9% of the variance in test performance.

**TABLE 1:** Correlations Matrix between the behavioral variables of the d2 test (CP, TOT, VAR, and Errors) (*n* = 61).

		**CP**	**VAR**	**Errors**	**TOT**
CP	Pearson Correlation	1	−0.279*	−0.631**	0.890**
	Sig. (2-tailed)		0.030	0.000	0.000
	N	61	61	61	61
VAR	Pearson Correlation	0.279*	1	0.411**	−0.099
	Sig (2-tailed)	0.030		0.001	0.447
	N	61	61	61	61
Errors	Pearson Correlation	−0.631**	0.411**	1	−0.411**
	Sig. (2-tailed)	0.000	0.001		0.001
	N	61	61	61	61
TOT	Pearson Correlation	0.890**	−0.099	−0.411**	1
	Sig. (2-tailed)	0.000	0.447	0.001	
	N	61	61	61	61

*Correlation is significant at the 0.05 level (2-tailed). **Correlation is significant at the 0.01 level (2-tailed). Bold numbers are significant correlations.

The original variables were grouped in three news components (see Table 2).

**TABLE 2:** Principal Component Analysis Matrix. CP, VAR, Errors, and TOT are the included variables, PC1, PC2, and PC3 are the components.

**Variables**	**Component**		
	**PC1**	**PC2**	**PC3**
CP	**31,506**	5, 859	1, 651
VAR	−13, 065	**25,360**	−10, 189
Errors	−22, 356	9, 598	**16,584**
TOT	**27,363**	13, 204	6, 783

*Extraction Method: Principal Component Analysis. Three component extracted. The component loadings are the correlations between the original variables and the components. Bold numbers represent stronger correlations.*

PC1: Selective attention and inhibitory control
PC2: Fluctuation rate
PC3: Errors

**FIGURE 3:**
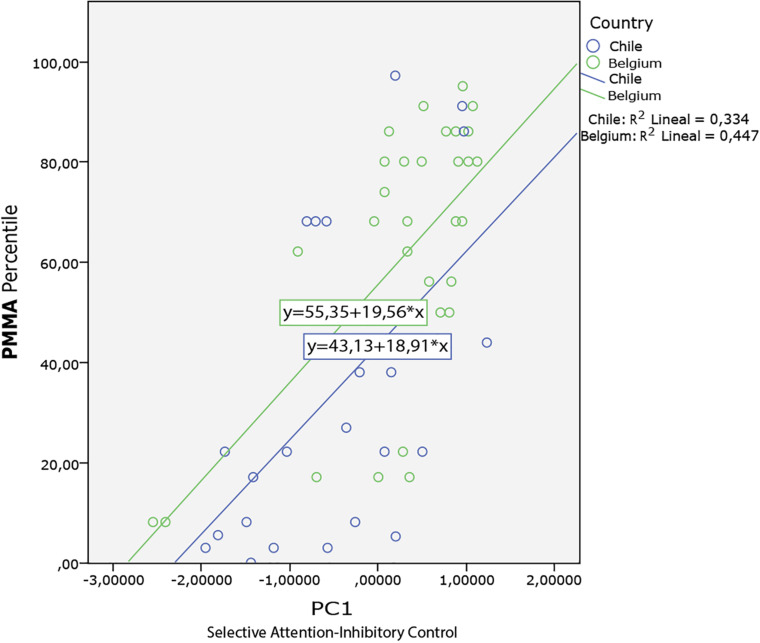
ANCOVA analysis. Geographical location as fixed factor Belgium (blue) and Chile (green) PC1as covariable and PMMA as dependent variable.

Then we did an ANCOVA analysis, with PMMA as the dependent variable, PC1, PC2, PC3 as covariables, geographical location, and musical background as fixed factors. For PC1 (*F* = 32.63; *p* < 0.001), PC2(*F* = 0.294; *p* = 0.590), and PC3 (*F* = 2,118; *p* = 0.151), Geographical location (*F* = 4.158; *p* = 0.046) and Musical Background (*F* = 0.003; *p* = 0.953). These analyses demonstrate that only PC1 (Selective attention and inhibitory control of attention) are related with audiation. Also we found that geographical location is significant for the relation between the attention and inhibitory control with audiation (see Figure 3).

## Discussion

In this study, we investigated the relationship between EFs and musical aptitude, focusing on audiation or the process through which sound becomes music and meaning is attributed to that music (Gordon, 2004). Audiation requires concentration on one musical pattern while simultaneously attending to or performing one or more other musical patterns (Gordon, 2007). To our knowledge, it has never been reported how EFs like sustained attention and inhibitory control could be correlated with audiation.

### Context and Musical Experience Influence

Because our study was conducted in two different urban schools, one in Belgium and the other one in Chile, we assumed that the context might influence the outcomes. Although both schools have similar characteristics, i.e., they are public (no fees) and non-profit, the Chilean and Belgium contexts could be quite different. Indeed, Chile has one of the highest income inequalities compared to other member countries of the Organization for Economic Cooperation and Development (OECD) and large differences in educational deprivation exist between children who come from high and low socio-economic status (SES; Escobar et al., 2018). Parents’ occupation and their level of education have a high impact on children’s behavior and attentional problems in 1st and 2nd grade children (Kalff et al., 2001). Another impactful factor is living in a deprived environment (Kalff et al., 2001). Also parents’ educational level has an impact on the development of EFs in children as an influence of environmental factors (Ardila et al., 2005). In a multicentric study children from Mexico, Puerto Rico, and Spain where the parents had a mean level of parental education (MLPE) >12 years obtained higher d2 TOT scores than children whose parents had a MLPE ≤12 years. They found that a child’s gender did not affect the d2 TOT scores for any country (Rivera et al., 2017). In line with this, we found lower scores in both CP in the d2 test and PMMA in Chilean children. Bivariate analysis indicated significant differences between Belgian and Chilean children in the total performance of the d2 test (TOT), the level of concentration (CP), and in the PMMA performance (rhythm), with lower percentiles in both tests. Moreover, the only variable that had an effect on performance in the d2 test and in PMMA was the geographical location, which was then confirmed with the ANCOVA analysis.

Furthermore, audiation has previously been correlated with other musical outcomes (Hanson, 2019). However, in contrast to other studies where early musical training (<7 years) was shown to have a positive effect on a rhythm synchronization task (Bailey and Penhune, 2012) or on auditory-evoked responses (between 7 and 13 years) (Putkinen et al., 2014), we did not find a significant effect of musical experience on sustained attention, cognitive flexibility or audiation. One possible explanation for the difference between our results and the results of Putkinen et al. could be that the 7 year old children in their studies had been playing instruments also for a short time, but had received formal musical instruction or if they have received informal musical instruction, have been exposed to musically enriched auditory environment (Putkinen et al., 2013).

### Selective Attention, Inhibitory Control and Musical Abilities

Our results indicate that scores on the audiation test could be explained by attentional levels and inhibitory control. Selective attention or executive attention is an endogenous, voluntary and top down process that allows one to choose voluntarily, to ignore stimuli, and to focus on others (Posner and DiGirolamo, 1998; Theeuwes, 2010; Diamond, 2013). Attention is significantly related to rhythm perception ability (Strait et al., 2011). Actually, children and adults with Attention Deficit Hyperactivity Disorder (ADHD) display difficulties in perceiving and reproducing event durations, in telling or reproducing the duration of visual and auditory stimuli and in comparing time intervals (Noreika et al., 2013). Also, impaired timing is associated with poor reading, attention and language (Noreika et al., 2013; Carr et al., 2014; Puyjarinet et al., 2017). Considering that audiation is, rather than being mere rhythm perception, defined as the cognitive process of hearing and comprehending musical sounds in terms of tonal and rhythmic contexts (Gordon, 1979), our results demonstrate that for audiation it is necessary to maintain the focus of attention and remain alert during relatively long periods of time.

Inhibitory control of attention or interference control at the level of perception (Diamond, 2013) allows to selectively attend or focus on what we choose and suppress attention to other stimuli, which is known as bottom up or automatic attention (Posner and DiGirolamo, 1998; Theeuwes, 2010; Diamond, 2013). In Diamond (2013), it has been proposed that errors seem to occur with the difficulty in inhibiting what might be termed “attentional inertia,” or the tendency to continue to focus attention on what had previously been relevant (Kirkham et al., 2003; Kloo and Perner, 2005, recently modeled by Chatham et al., 2012). This is of interest with regard to completing the PMMA test, the results of which have been previously linked to personality aspects of concentration and perseverance (Nijs, 2012). Gordon proposed that holding patterns in mind while simultaneously updating WM with the relevant tonal and metrical context requires to inhibit attention to all irrelevant details (Gordon, 2007). The results of our study suggest a close relationship between audiation and these EFs.

Finally, associations between musical aptitude scores and other variables have regularly been found (Schellenberg and Weiss, 2013). Indeed, music aptitude is not only associated to teachers’ or parents’ evaluation of children’s musicality (Drake, 1954; Tarrell, 1965; Harrington, 1969; Davies, 1971; Young, 1972, 1976), but also to language (e.g., phonological awareness, reading ability), mathematics (e.g., arithmetic abilities), personality (Nijs, 2008; Swaminathan and Schellenberg, 2018) and general cognitive skills (see Schellenberg and Weiss, 2013 for an overview). With regard to the latter, music aptitude has been associated with general cognitive abilities (e.g., Norton et al., 2005) and with specific aspects of cognition, such as WM (Wallentin et al., 2010), mental speed (Gruhn et al., 2003), or spatial abilities (Nelson et al., 1992). Very often such associations are discussed within the context of music training and how it affects cognitive development. However, such a causal link has only been shown on the basis of a small amount of evidence while much more evidence exists in favor of shared neural resources for music and other cognitive abilities (Magné and Gordon, 2017).

### Methodology

The aim of our study was to investigate the relationship between EFs and musical audiation. This study arose from our interest in studying the correlation between rhythmic synchronization skills and cognitive functions such as attention, inhibitory control and cognitive flexibility. As rhythmic synchronization is related to children’s musical aptitude, we introduced a standardized test on music aptitude, more specifically the Rhythm Subtest of the PMMA. To test the EFs of the children, we used the standardized test d2.

Using such tests with younger children always involves a risk with regard to reliability due to the fact that younger children have greater difficulties in completing long batteries of cognitive tests (Karr et al., 2018). Therefore the PMMA was deemed appropriate. It is the only brief, longitudinally valid music aptitude test for Grades K through 3. It is a very child friendly test. Using pictorial representations, it does not require the ability to read. In addition, no prior music instruction is required to take the test. However, some concerns with regard to the test need to be taken into account. For example, Yang (2002) reported that children of the first grade from Taiwan scored significantly lower than the published norms on the PMMA Tonal subtest and non-significantly lower on the Rhythm subtest (Yang, 2002; Stamou et al., 2010). These results are important for our study, since in our study we perform only the rhythm subtest. Validation of the PMMA for Chilean children could be a next step. A limitation of our study was the different way of administering the PMMA in the two countries. This was necessary for practical reasons, asked for by the school in Chile.

The d2 was used because it measures different cognitive functions we were interested in. It could be argued that, because the different scores measured by the test were correlated with each other, and for that reason may not be interpreted as a different construct. However, it has been well documented that the d2 gives a reliable measure of selective attention (Moore and Malinowski, 2009; Lozano et al., 2015). Also, to evaluate the construct validity of the d2 test, a factorial analysis was conducted between the d2 and Stroop-test, and d2 scores were submitted to two factors, factor 1 correlated with Stroop-test, especially with word interference-color, which is a measure of concentration, distraction and inhibition (Brickenkamp and Zillmer, 1998). This means that d2 is reliable to measure selective attention and inhibitory control.

## Conclusion

In this study, we have linked cognitive functions to audiation skills. To our knowledge, this has not been previously addressed in an empirical study. Our findings suggest that both human faculties are strongly related to each other.

Given these results, we believe it is important for future studies on human interaction with music and music learning to take into account this relationship. As Gordon points at the different steps in music processing and as we have empirically shown, we propose that it is important to take into account people’s variability in EFs capacity when studying cognitive processes involved in music. For example, many studies investigate the role between movement and learning, assuming that the bodily involvement reflects the cognitive processing of the music. Here, elements such as attention, inhibitory control or cognitive flexibility play an important role. However, these are never taken into account. Our study suggests that it is important to do this to better understand interaction with music.

Our results may also stimulate further research on music cognition in relation to more general cognitive skills.

## Data Availability Statement

The datasets generated for this study are available on request to the corresponding author.

## Ethics Statement

The studies involving human participants were reviewed and approved by Research Ethics Committee from the Faculty of Arts and Philosophy, Ghent University, Research Ethics Committee from the University of Santiago, Chile N°: 314/2019. Written informed consent to participate in this study was provided by the participants’ legal guardian/next of kin.

## Author Contributions

NG, LN, LK, and KO planned and conducted the study. NG, LN, KO, and NS programed and applied the tests at the schools. NG and AR-H analyzed the data. NG, LN, and LK wrote the first draft of the manuscript. All authors contributed to the compilation of the manuscript and read and approved the submitted version.

## Conflict of Interest

The authors declare that the research was conducted in the absence of any commercial or financial relationships that could be construed as a potential conflict of interest.

## Footnotes

1 http://www.giml.org

## Supplementary Material

TABLE S1 Supplementary Material d2 percentiles and PMMA percentiles. Geographical location, Gender and Musical background.
